# Arsenic in the water and agricultural crop production system: Bangladesh perspectives

**DOI:** 10.1007/s11356-022-20880-0

**Published:** 2022-05-26

**Authors:** Arifin Sandhi, Changxun Yu, Md Marufur Rahman, Md. Nurul Amin

**Affiliations:** 1 Department of Biology and Environmental Science, Faculty of Health and Life Sciences, Linnaeus University, 391 82 Kalmar, Sweden; 2 Department of Crop and Soil Sciences, Washington State University, Pullman, WA 99164-6420 USA; 3 Breeder Seed Production Centre, Bangladesh Agricultural Research Institute, Debiganj, Panchagarh-5020, Bangladesh; 4 Bangladesh Institute of Research and Training On Applied Nutrition, Rangpur Regional Station, Pirgonj-5470, Rangpur, Bangladesh

**Keywords:** Arsenic, Health, Water, Irrigation, Arsenic accumulation, Mitigation policies, Arsenic removal, Food chain

## Abstract

The presence of high levels of carcinogenic metalloid arsenic (As) in the groundwater system of Bangladesh has been considered as one of the major environmental disasters in this region. Many parts of Bangladesh have extensively reported the presence of high levels of arsenic in the groundwater due to both geological and anthropogenic activities. In this paper, we reviewed the available literature and scientific information regarding arsenic pollution in Bangladesh, including arsenic chemistry and occurrences. Along with using As-rich groundwater as a drinking-water source, the agricultural activities and especially irrigation have greatly depended on the groundwater resources in this region due to high water demands for ensuring food security. A number of investigations in Bangladesh have shown that high arsenic content in both soil and groundwater may result in high levels of arsenic accumulation in different plants, including cereals and vegetables. This review provides information regarding arsenic accumulation in major rice varieties, soil-groundwater-rice arsenic interaction, and past arsenic policies and plans, as well as previously implemented arsenic mitigation options for both drinking and irrigation water systems in Bangladesh. In conclusion, this review highlights the importance and necessity for more in-depth studies as well as more effective arsenic mitigation action plans to reduce arsenic incorporation in the food chain of Bangladesh.

## Introduction

Arsenic (As), the 20th most abundant naturally occurring element in the earth’s crust (Mandal and Suzuki 2002), occurs ubiquitously in the environment and is highly toxic. Elevated levels of As in soils, waters, and plants pose a long-term negative effect on the ecosystem. Long-term exposure to As could cause a number of chronic diseases, including but not limited to skin lesions, skin cancer, kidney, lung, bladder, hypertension, cardiovascular diseases, and DNA methylation (Rahman et al 2015; Smith et al. 2000; Saha and Rahman 2020; Ljung et al. 2011). Strongly elevated levels of arsenic (< 0.5– > 4600 µg/L) have been found in the groundwater systems in several regions of Bangladesh and are considered to be one of the major environmental disasters of this South-East Asia region (Huq et al. 2020; Rahman et al. 2019b; Sandhi et al. 2017; Smith et al. 2000). Arsenic poisoning (via e.g., drinking water) poses significant public health concerns for Bangladesh and is estimated to affect about 35–77 million inhabitants throughout the country (Rahman et al. 2018; Smith et al. 2000). Along with As-rich groundwater, another major arsenic exposure pathway is the consumption of rice grains that have been irrigated regularly with As-rich groundwater (Rahman et al.2019a; Williams et al. 2006). Both geogenic processes and anthropogenic activities are responsible for the arsenic that originates in the environment system (Saha and Rahman 2020; Sandhi et al. 2018). The arsenic content found in different food grains and vegetables is gathering attention as a source for potential global health risk due to the possibility of people in arsenic non-contaminated regions being exposed to arsenic via the global food trade (Rahman et al. 2018). In this review, we have assessed the existing articles dealing with arsenic occurrence, distribution, and accumulation exclusively related to- and investigated in Bangladesh. Therefore, the main purpose of this review is to coalesce the information and literature with special focus on the problem of arsenic contamination in the soil–water system and its content in the major food crops in Bangladesh.

## Arsenic chemistry in soil and groundwater system

Arsenic concentration in terrestrial soils typically varies from 1.5 to 3 mg/kg, depending on the origin of the soil (Heikens et al. 2007; Mandal and Suzuki 2002). Arsenic has four oxidationon states, − 3, 0, + 3, and + 5, with the last two being most common in the surface environment. Redox potential and pH are the two key factors regulating As speciation in the soil medium. In general, arsenate (As^5+^) is the major species in the aerobic or oxygenated soil, whereas arsenite (As^3+^) prevails in the anaerobic or submerged soil (Zhao et al. 2010). Arsenite and arsenate commonly hydrate as arsenious acid and arsenic acid, with different charges depending on the solution’s pH (Lizama et al. 2011). For instance, arsenite and arsenate commonly occur as uncharged species (H_3_AsO_3_ and H_3_AsO4) under normal and acidic conditions, but as negatively charged species (H_2_AsO_3_^−^, HAsO_3_^2−^, and AsO_3_^3−^ for arsenite; H_2_AsO4^−^, and HAsO4^2−^ for arsenate) at high pH (> 6) conditions (Lizama et al. 2011; Yu et al. 2014)). Besides those inorganic arsenic species, Monomethylarsenic acid, or MMA [CH_3_AsO(OH)_2_], and dimethyl arsenic acid, or DMA [(CH3)_2_AsO(OH)], are also present in the soil both at low and high concentrations (Zhao et al. 2010).

Elements such as Fe (iron), P (phosphorus), S (sulfur), and Si (silicon) play a vital role for both As mobilization and uptake in plant species. Among those elements, Fe redox chemistry plays a significant role in arsenic behavior in the soil medium. FeOOH acts as the main sorbent for both inorganic arsenic species (arsenate and arsenite) (Takahashi et al. 2004; Heikens et al. 2007). For arsenic released into the soil, both the type of soil and the presence of oxygen are the two vital factors that could stimulate FeOOH to release arsenic species into the soil medium. (1) FeOOH predominantly in the clayey soil compared with sandy soil, therefore the arsenic content in the clayey soil is relatively higher than sandy soil (Fitz and Wenzel 2002). Even though there is a higher concentration of arsenic present in the clayey soil, the clayey soil is less toxic due to arsenic being more tightly bonded with clayey soil than with sandy soil. (2) The oxygenic condition of soil has a significant effect on the dissolving capacity of FeOOH. Under anoxic conditions, FeOOH can be quickly reduced, releasing arsenate into the soil medium which is ultimately reduced to arsenite, whereas under oxic conditions, FeOOH is moderately insoluble and considered as a sink of arsenic (Heikens et al. 2007). Meanwhile, a recent investigation in the river Meghna floodplain (central region of Bangladesh) area has found that reductive dissolution of Fe and Mn oxyhydroxides was the principal process of arsenic release in the groundwater system (Saha and Rahman 2020).

Besides Fe, P also plays an essential role for arsenic absorption and accumulation in plants as P is considered to be an analog of arsenate (Zhao et al. 2010). Phosphorus competes with inorganic arsenic species of both arsenate and arsenite for adsorption sites at Fe oxide surfaces ( Hossain et al. 2009). As such, it has been shown that the addition of phosphate could lead to remobilization of sorbed arsenate from exchangeable sites and thus enhance the solubility, bioavailability, and movement of arsenate from soils (Abedin et al. 2002). It was previously speculated that the extensive groundwater-based irrigation and application of P-based fertilizer in the crop fields could initiate arsenic release into the groundwater system in Bangladesh. However, it was discovered that it is not present in several arsenic-contaminated sites for example in the central east part of Bangladesh (Acharyya et al. 2000; Saha and Rahman 2020).

Sulfur can affect arsenic speciation and mobilization in the soil medium through two ways: (1) sulfide produced from bacterially mediated sulfate reduction can reduce and trap arsenic as As-sulfide minerals in the sediment (e.g., orpiment (As_2_S_3_) and realgar (AsS)) and (2) sulfide can also reduce As-bearing iron oxides, liberating the sorbed arsenic (Fischer et al. 2021; Saalfield et al. 2009). The seasonal variation in Bangladesh can also influence the arsenic leaching from As-sulfide mineral sources. Polizzotto et al. (2005) found that the throughput of the dry season, e.g., sulfide minerals that are oxidized, leading to the repartitioning of arsenic into ferric hydroxides which is continued by reductive dissolution of iron and arsenic during the consequent wet season. Therefore, cyclic redox conditions in the near-surface sediments have an influence toward arsenic mobilization in groundwater system in Bangladesh.

## The occurrence of arsenic in Bangladesh

Arsenic contamination of groundwater in Bangladesh has been attributed to two processes: (1) oxidation of arsenic-rich pyrite by the presence of atmospheric oxygen due to lowered groundwater tables and (2) reduction of iron oxyhydroxide coupled to organic matter degradation during high water flow conditions (Nickson et al. 1998; Rahman et al. 2016). To understand the mobilization of global arsenic contamination, a number of theories have developed that include reductive dissolution, alkali desorption, sulfide oxidation, and geothermal activity (Ravenscroft et al. 2009). Among those, reductive dissolution has been considered to be major reason for arsenic mobilization in Bangladesh due to the humid and tropical climate present in the alluvial Bengal Delta zone (Raessler 2018).

Using surface waters as drinking water was not safe for the mass population in Bangladesh especially in 70–80s. Prior to this, both adults and children were affected by a number of gastrointestinal diseases due to the frequent consumption of bacteria-contaminated surface waters and are considered to be one of the major reasons for the number of water-borne diseases and mortality. The presence of those microorganisms in the surface water sources in Bangladesh have been an influencer for use of tubewell-based water. During the 1970s, both the United Nations Children’s Fund (UNICEF) and Department of Public Health Engineering (DPHE), Bangladesh, jointly started installing tubewells for increasing coverage of supply and access to safe drinking water in Bangladesh (Smith et al. 2000; Gamble et al. 2007). Then in the early 1990s, arsenic contamination was discovered in the groundwater of Bangladesh and was considered to be a major environmental catastrophe (Khan et al. 1997). In 1993, the presence of high levels of arsenic in tubewell water in Bangladesh was confirmed in 44 districts and arsenicosis was detected in 26 districts of them (Khan et al. 1997). According to Raessler (2018), out of 11 million tubewells, approximately 3 million of them contained more than 10 µg/L arsenic (10 µg/L arsenic has recommended as maximum safe water limit according to WHO (World Health Organization).

The presence of high levels of arsenic in the food grains, especially in the rice and vegetables grown in Bangladesh, got exposure at the beginning of this millennium (Meharg and Rahman 2003). Rice is considered to be a staple food in this region. It is well established as one of the major agronomic crops grown in Bangladesh and it requires a high level of irrigation for optimum yield. To fulfil this vast irrigation demand for rice cultivation in Bangladesh, the farmers source more of their irrigation water from groundwater (39.9%) than from surface water (0.2%) sources (Shah et al. 2006; Hussain et al. 2021). Therefore, frequent groundwater-based irrigation increases arsenic load in the agricultural fields, and that in turn increases arsenic availability and load in the rice cultivars grown in Bangladesh. Seasonal variation and frequent flooding also play an important role for arsenic deposition in the agricultural soil in Bangladesh. In the topsoil, arsenic concentration increases during irrigation period and later during the monsoon flooding season, arsenic content become reduced (Saha & Ali 2007; Chowdhury et al. 2021). It is estimated that due to the groundwater-based irrigation, arsenic load in the soil could reach annually 4 kg As/ha in the soil, whereas 0.5–2.5 kg As/ha were released from the soil during the monsoon flooding process (Dittmar et al. 2010a).

## Arsenic in water system

### Drinking water

The network of tubewells in Bangladesh have operated since 1940, and later expanded in the 1970s, to provide safe drinking water sources for the population, but in 1993, high arsenic content was found in the tubewell drinking water in Barughuria Union, Nawabgonj district, for the first time by the DPHE (Rahman 2002; Smith et al. 2000). During the late 1990s, one of the earliest investigations of arsenic content of tubewell water in Bangladesh reported arsenic presence in the tubewell water samples. The investigation analyzed water samples from 3490 tubewells and found that 28.1% and 21.9% of the surveyed tubewells contained As concentration (> 50 µg/L) and (10–50 µg/L) respectively (Khan et al. 1997). Later on, a systematic nationwide arsenic survey was conducted by both BGS (Bangladesh Geological Survey) and DPHE, and found that 27% of the surveyed shallow tubewells (< 150-m depth) had arsenic concentrations higher than the Bangladesh drinking water standard (50 µg/L As) (Ahmed et al. 2004). In 1999, two arsenic mitigation pilot projects (including tubewell water testing, mitigation option feasibility, and public awareness schemes) were launched by the NGO (non-government organization) BRAC (Bangladesh rural development committee) in Bangladesh (Chowdhury et al. 2000). Later on, a number of Bangladesh based NGOs (e.g., NGO Forum for Drinking water & Sanitation, Proshika) initiated arsenic mitigation programs in different areas in Bangladesh (Milton et al. 2012). However, even after such mitigation options were implemented in different arsenic affected areas in Bangladesh, the inhabitants still lack awareness about arsenic, as well as, lack interest to identify safe drinking water sources. A comparative study (blanket surveys) 12 years apart in Bangladesh have found that 66% of 2041 tubewells were unsafe, but were still used for drinking and cooking purposes (van Geen et al. 2014).

Meanwhile, a number of research investigations regarding arsenic content in the drinking water and its health effect were initiated in different parts of the country during the late 1990s. Those investigations have indicated that along with skin lesions, arsenic exposure through drinking water could increase diabetic mellitus, hypertension, and lung cancer (ninefolds higher) in the human body (Yunus et al. 2011; Argos et al. 2014). Furthermore, several chronic respiratory symptoms have been increasingly found in children due to their early life exposure to arsenic (Smith et al. 2013). A recent study in Bangladesh has also found that consumption of low to moderate level of arsenic (50 to 150 µg/L) through drinking water could increase hyperglycemia especially in female individuals (Paul et al. 2019). Moreover, a study conducted in Nawabganj municipality, Chapai-Nawabganj (a north-western district in Bangladesh), has reported that average arsenic concentration in the groundwater of that area reached 48.81 mg/L (Selim Reza and Jean 2012). Arsenic ingestion during pregnancy has significant health effects, for example the maternal urinary arsenic level during pregnancy of the mothers correlated with reduced thymus size and T-cell proliferation in the newborns (Welch et al. 2019; Ahmed et al. 2012; Raqib et al. 2009). Besides chronic disease-related problems, arsenic can cause a problem at the genetic level in the human body as arsenic exposure through drinking water has a strong influence on cellular level immunity and DNA methylation in the children that have been raised in the arsenic affected areas in Bangladesh (Ahmed et al. 2014; Broberg et al. 2014). The aforementioned studies show that arsenic exposure through drinking water play an important role for wellness of children and females especially in highly arsenic-contaminated areas.

### Irrigation water

Being an agricultural-based country, the cultivation of cereal crops is considered to be one of the major agricultural activities in Bangladesh. The advancement of rice HYVs (high yielding varieties) require high levels of irrigation during the dry season for their production. Boro rice (high yielding rice varieties) cultivation has increased since 1970 and cover nearly 20% of all of Bangladesh which is equivalent to almost 45% of the total cultivable land areas of Bangladesh (Harvey et al. 2006). However, the high volume of irrigation practices is performed by using surface water and also shallow and deep tubewell sources. A report showed that during the 2007–2008 dry season, approximately 1.3 million units of shallow tubewells had been used to irrigate 3.2 million ha of land in Bangladesh (Hossain 2009). Those shallow tubewells play a major role for deposition of high levels of arsenic in the topsoil. The number of investigations about arsenic content in irrigation water were relatively fewer compared to the arsenic content in drinking water in the 1990s. A study found that arsenic concentrations could reach up to 20–30 mg/kg in soils that have been irrigated frequently with groundwater (Meharg and Rahman 2003). This added arsenic (both arsenate and arsenite) from groundwater-based irrigation could be temporarily sunk in the topsoil, and that then could be easily released into the water-soil system and embedded in the food chain system (Martin et al. 2007). However, a number of factors play essential roles for As retention in the agricultural soil such P, Fe content, distance from irrigation channel, duration, and irrigation water condition (flowing or static) (Polizzotto et al. 2013; Dittmar et al. 2010b; Duxbury and Panaullah 2007). Meanwhile, several studies have confirmed that arsenic content in the irrigation water in Bangladesh increased As content in the paddy soil, rice grain and also decreased rice grain yield (Huhmann et al. 2019; Khan et al. 2009, 2010; Panaullah et al. 2009).

## Arsenic in food crops/agriculture

### Cereal crops

Most of the crop cultivation in Bangladesh have been greatly dependent on groundwater-based irrigation management due to lack of surface water sources. Next to drinking water, a number of studies have already indicated that the consumption of food crops cultivated with As-rich groundwater is another crucial pathway of arsenic accumulation into the human body in Bangladesh (Panaullah et al. 2009; Sinha and Bhattacharyya 2015; Tani et al. 2012; Alam et al. 2019). This arsenic exposure is due to growing food crops in arsenic-contaminated soil and/or irrigated with arsenic-contaminated groundwater. In Bangladesh, rice is one of the major agronomic crop varieties, covering up to 75% of total agricultural land and requiring 83% of irrigation water (Sandhi et al. 2017). The use of As-contaminated irrigation water in Bangladesh has been found to be the main cause for the accumulation of this carcinogenic metalloid in the food crops (Das et al. 2009).

The accumulation of arsenic in the rice grains is strongly correlated with As concentrations in the soils (Suriyagoda et al. 2018a). Similar tendencies have been identified for arsenic uptake in plant grown in the areas with a high concentration of As in soils and irrigated with arsenic-rich groundwater (Kabir et al. 2016). Meharg and Rahman (2003) has reported that total arsenic concentrations in rice grains from Bangladesh range between 0.058 and 1.835 mg/kg, similar to what reported for raw rice grown in the USA (0.2–0.46 mg/kg) and in Taiwan (0.063–0.2 mg/kg). Therefore, arsenic loading in the rice grain depends greatly on both the arsenic-contaminated ground water-based irrigation system and the continuously loaded arsenic in the top soil in the agricultural lands. Meanwhile, arsenic speciation (ex; inorganic and organic arsenic species) in the rice grain have observed different trends which could be a possible outcome from arsenic content in particular locations. For example, US-grown rice contains mainly DMA compared to the rice varieties grown in Bangladesh which contain mainly inorganic arsenic and DMA is considered as less toxic related to inorganic arsenic species (Duxbury et al. 2009). For instance, Duxbury et al. (2009) has found that arsenic in Bangladeshi rice varieties contain a mixture of about 80% inorganic forms and 20% of organic forms. A survey about arsenic exposure through rice consumption in Marua village, Jessore, Bangladesh, has found that an adult consumed 220 µg arsenic per day from a food source, and rice alone contributed 86% of it (Tani et al. 2012). Similar to this study, Geng et al. (2006) has also shown that the amount of As ingested through rice consumption is about 0.08 µg g^−1^ and is very similar to the drinking water As level of 10 µg L^−1^. However, arsenic-related human health risks and their analysis through rice consumption not only depend on the rice varieties, arsenic content in the soil and irrigation water of those cultivated rice fields, and their arsenic AF (accumulation factor, metal/metalloid concentration ratio of plant to soil). A field study conducted in Matlab, Bangladesh, showed that arsenic content in the de-husked grain of hybrid rice cultivars was low compared to the local aromatic rice cultivar and that it could reduce health risk through rice consumption; however, they have a higher accumulation factor than the local rice varieties (Sandhi et al. 2017). Therefore, the rice cultivars high arsenic AF could increase arsenic ingestion in the human food chain more if they have been cultivated in As-rich environments (soil and groundwater).

Application of As-rich irrigation water and the soils, both are accountable for arsenic transfer and uptake in different parts of rice plants, including straw, husk, and grain in Bangladesh (Kabir et al. 2016). The arsenic content in rice varies depending on the type of rice cultivar, the place where it was cultivated, and how it was processed. In Table 1, the difference between arsenic content in the rice husk and grain parts of common rice varieties grown in Bangladesh is shown. Previous datasets have included scientific information pertaining to rice cultivar investigations from the fields of or collected grains, and later laboratory grown conditions, exclusively from Bangladesh in last 20 years, not any other regions or countries. Meanwhile, a number of studies regarding As content in rice grain have been performed in Bangladesh, but most of them have focused on household rice sample collection (without accompanying soil and irrigation water samples). Therefore, it is not easy to locate and co-relate the location of the rice field precisely and in our review, we have omitted those data. A number of studies have found that the Brown rice contains higher concentrations of arsenic than white rice (Norton et al. 2010; Torres-Escribano et al. 2008; Williams et al. 2005). Moreover, changes in arsenic content may occur during the preparation of food where cooking water could play a significant role; cooking or preparation of rice with arsenic non-contaminated water could reduce the arsenic content of the rice (Cubadda et al. 2003). The cooked rice lowers 17% and 46% of the As exposure and risk in terms of Miniket and Kataribhogh rice (respectively, common names) (Sekine et al. 2017). On the other hand, Sandhi et al. (2017) has found that the de-husked grain of local aromatic rice (kalijira cultivar) had higher (0.09–0.21 mg kg^−1^) content, whereas the HYV (high yield varieties) rice contained less arsenic concentration. Another study has found that the outer fraction of rice (husk) might act as a translocation barrier for As absorption by rice grain (Kabir et al. 2016). The International Codex Alimentarius has revised the standard for maximum levels for inorganic arsenic in husked rice grain (0.35 mg kg^−1^) and polished rice grain (0.20 mg kg^−1^) in 2019 (FAO and WHO 2019). Arsenic levels in Boro season rice are higher than Aman season rice, and it might co-relate with the increased irrigation with As-rich groundwater in the Boro rice and the mitigation recommendation was to reduce groundwater-based irrigation frequency (Duxbury et al. 2003; Meharg and Rahman 2003; Williams et al. 2006).

**Table 1:** Total arsenic concentration (µg g^−1^ DW) in grain and husk of different rice cultivars of high yield varieties (HYV) and local aromatic cultivars grown in Bangladesh. This dataset includes exclusively those works either done in the field or pot cultivation in Bangladesh

Rice cultivars	Rice type	Cultivation media	De-husked rice grain	Husk	Rice grain	References
BRRI dhan 33	HYV	Pot culture	0.70–0.20	6.21–0.295	–	Hossain et al. 2009
BRRI dhan 28	HYV	Field	0.6 ± 0.0	1.7 ± 0.1	–	Rahman et al. 2007
BRRI hybrid dhan 1	HYV	Field	0.7 ± 0.2	1.9 ± 0.1	–	Rahman et al. 2007
BRRI dhan 29	HYV	Field	0.59–0.22	2.48–1.20	–	Khan et al. 2010
BRRI dhan 33	HYV	Field	0.81–0.22	2.24–1.32	–	Khan et al. 2010
BRRI dhan 29	HYV	Field	–	–	0.65 ± 0.02	Talukder et al. 2011
BRRI dhan 10	HYV	Field	–	–	0.31 ± 0.02	Williams et al. 2006
BRRI dhan 11	HYV	Field	–	–	0.21 ± 0.0	Williams et al. 2006
BR 11	HYV	Field	–	–	1.84–1.74	Meharg and Rahman 2003
Bina Dhan 6	HYV	Field	–	–	0.18 ± 10	Islam et al. 2017
Bina Dhan 14	HYV	Field	–	–	0.30 ± 16	Islam et al. 2017
Kheali Boro	Local	Field	–	–	0.36 ± 16	Islam et al. 2017
Kalijira	Local	Field	–	–	0.18 ± 0.3	Williams et al. 2006
Nayanmoni	Local	Field	–	–	0.27 ± 0.02	Williams et al. 2006
Swarna	Local	Field	–	–	0.096	Meharg and Rahman 2003
Kalijira	Local	Field	0.21–0.09	0.134–0.065	–	Sandhi et al. 2017

### Horticultural crops

After the observation of high levels of arsenic in the groundwater system and irrigation water of Bangladesh, the concern for arsenic content in vegetables has also increased. One of the very initial studies found arsenic concentrations in different kinds of vegetables, including bottle gourd (*Lagenaria siceraria*), taro (*Colocasia esculenta*), and eddoe (*Colocasia schott*), and at the time of publication it was below the arsenic PTWI (provisional tolerable weekly intake) of 0.015 mg kg^−1^ body weight available (Alam et al. 2003). In 2004, a study reported that total arsenic concentrations in arum leaves (*Colocasia antiquorum*), potatoes (*Solanum tuberosum*), and water spinach (*Ipomoea reptans*) were 0.09–3.99 mg/kg, 0.07–1.39 mg/kg, and 0.1–1.53 mg/kg, respectively (Das et al. 2004). They have also demonstrated that growth in aquatic conditions and proximity to nearby an As-contaminated tubewells are the reasons for high arsenic content in both arum leaves and water spinach. Another study found that arsenic concentrations in vegetables imported from Bangladesh were 2-–100-fold higher than vegetables that were grown in UK (United Kingdom), EU (European Union), America, and Croatia (Al Rmalli et al. 2005). A study found that crops within the Araceae family (such as eddoe (*Colocasia schott*) and taro (*Colocasia esculenta*)) contained higher levels of arsenic in Feni district (Southeast part of Bangladesh) and application of hand or shallow tubewell-based irrigation could be a possible reason for the elevated levels of arsenic in these crops (Karim et al. 2008).

In recent years, an investigation of 13 different vegetable species grown in Bangladesh found high arsenic concentration (0.52 mg/kg fresh weight) and AF in Bean (*Phaseolus vulgaris*) and the possible reasons for this high arsenic content includes, both As-rich irrigation water and As-containing pesticide applications (Islam et al. 2016). Among different vegetable crops, leafy vegetables (lettuce) and bean vegetables have a strong tendency for higher arsenic accumulation and translocation in their above-ground parts (Greger et al. 2015). Although there is no global standard for the maximum arsenic limit in the vegetables, health agencies from several countries recommended 0.1 mg/kg FW (fresh weight) arsenic in the vegetables (Mcbride 2013). The number of investigations pertaining to arsenic content in vegetables was relatively fewer compared to the cereal crops, but relatively high arsenic translocation mechanism in vegetable crops demand more attention in order to reduce arsenic accumulation in the human body.

## Arsenic remediation technologies for groundwater and irrigation

When compared to the number of investigations about health effects of As, potential arsenic removal from drinking water has been rarely studied. Different arsenic removal and filtration technologies used in Bangladesh were summarized in Table 2. Since the beginning of this millennium, Bangladesh has started developed and has tested different technologies and methods to mitigate arsenic contamination in the groundwater system. A modified filtration technique namely a simpler 3 pitcher (locally also known as kolsi) has applied and successfully reduced arsenic concentrations in the filtrated water below the WHO As drinking water standard (10 µg/L) under laboratory conditions (Khan et al. 2000). Meanwhile, SIDKO (granular ferric hydroxide-based technology) has been introduced in Jhikargaccha Upzila for household water filtration, but it has a number of weakness, including high cost, low water flow rate, and intensive management (Chowdhury et al. 2000). Another water filtration technology Alcan (enhancedactivated alumina) has shown potential as it could efficiently reduce As, Mn, and Fe from the water system. However, it suffers from a high risk of bacterial contamination and less user-friendly structure (Jalil and Ahmed 2001).

**Table 2:** A summary of different techniques for mitigating arsenic contamination in the groundwater and agricultural fields in Bangladesh

Name of technologies	Tested medium	Location(s)	Uses	Reference
SIDKO (granular ferric hydroxide-based technology)	Groundwater	Jhikargaccha Upzila	Household	Chowdhury et al. 2000; Jones et al. 2008
Alcan (enhanced activated alumina)	"	Narayangonj District	Household	Jalil and Ahmed 2001
Improved dug well	"	Pabna District	Household	Joya et al. 2006
SONO filter (composite iron matrix)	"	16 different Districts	Household, Primary school premises	Hussam and Munir 2007
Arsenic removal filter (ARF)	"	3 subdistricts in Khulna division	Household	Shafiquzzaman 2017
Sub-surface arsenic removal (SAR)	"	Comilla	Household	Kundu et al. 2018
Pond sand filter	Surface water	Dacope Upzila	Household	Harun and Kabir 2013
Rainwater harvesting	Condensation of atmospheric water vapor	Dhaka Division	Household	Islam et al. 2010
Jute mushed irrigation channel	Irrigation water	Dhaka Division	Rice cultivation	Polizzotto et al. 2015
AWD (alternative wetting and drying)	Irrigation water	Mymensingh district	Rice cultivation	Norton et al. 2017a, b

A number of pilot-scaled technologies, such as improved dug well, pond sand filter, SONO filter, arsenic removal filter (ARF), sub-surface arsenic removal (SAR), and rainwater harvesting have been tested in different parts of Bangladesh during 2006–2017 (Hussam and Munir 2007; Milton et al. 2012; Kundu et al. 2018). Among these technologies, SONO filter got a lot of attention, because it has low economic cost and can be locally manufactured. It was patented in 2002 and 30,000 filter units were deployed all-over Bangladesh, but then slow water flow rate made it less popular (Hanchett et al. 2011; Hussam and Munir 2007). Moreover, other studies have also found that SONO filters could effectively remove arsenic for up to 8 years without any replacement (Neumann et al. 2013). The color of sediment could help to detect the safe drinking water sources during tubewell installation process. This sediment color detection method was developed in a prominent arsenic hotspot area named Matlab upzilla in Bangladesh, where the sediment color (during tubewell drilling processes) used for the identification of potential low arsenic aquifers in arsenic-contaminated areas, especially by the drillers involved for tubewell implementation processes (von Brömssen et al. 2007). Meanwhile, the SAR technique for arsenic mitigation from the groundwater sources has got attention in number of arsenic-contaminated locations on the Asian continent including Bangladesh also (Kundu et al. 2018).

It has been reported that approximately 106,939 arsenic mitigation options have implemented in different parts of Bangladesh e.g., installation of dugwells, pond sand filters, rainwater harvesters, deep tubewells, arsenic iron removal filters and so on (Milton et al. 2012). Meanwhile, another survey-based study (1268 Bangladeshi households) has also found the most acceptable options for safe drinking water in Bangladeshi rural inhabitants (high score: piped water system, household filter system, and deep tube well water), (medium score: community arsenic removal technologies, pond sand filter, rainwater harvesting), and (low score: dug wells and well sharing) (Inauen et al. 2013). In addition, for reduction of arsenic exposure through drinking water, the Government of Bangladesh (GOB) initiated a piped national-scale water system, financed by the World Bank during 2012–2017, supplying “clean” water to about 21,802 household (IEG Review team 2018). Implement of national-scale piped water systems could be the ultimate solution for reduction of arsenic contamination in drinking water in Bangladesh, as suggested by (Jamil et al. 2019).

In contrast to the reduction of arsenic content in drinking water sources, arsenic mitigation options/technologies for irrigation water were less tested/implemented in Bangladesh. A study found that a jute fiber mesh-based irrigation channel structure could reduce arsenic loading in rice fields in Bangladesh (Polizzotto et al. 2015). A study has found that the alternation of irrigation patterns like the permanent raised bed (PRB) could reduce arsenic content in the rice grain (Talukder et al. 2011). Besides those, it has been shown that the alternative wetting and drying (AWD) method-based rice cultivation could reduce arsenic content in the shoot (up to 26%), but also increased Cd (cadmium), Cu (copper), and Mn (manganese) contents in the rice plants (Norton et al. 2017a, b). Several agronomic and biotechnological strategies have suggested methods for reduction of arsenic content in the rice grain including the use of surface water (pond, lake, canal, river) or water from deep tubewells, modification of rice genotype through bioecological tools, and silicon fertilizer application (Das et al. 2020; Suriyagoda et al. 2018b). A study has found that rice genotype contributes 9–10% of the grain arsenic concentration in 38 BRRI (Bangladesh Rice Research Institute)-released rice varieties (Ahmed et al. 2011). Another study has also suggested that genetic variation plays a vital role in the rice grain arsenic content (Norton et al. 2012). Therefore, rice breeding has a strong potential for reducing grain arsenic content by identifying responsible genes for low arsenic content in the new rice cultivars.

Meanwhile, the post-harvest management of rice grain also plays a vital role for reduction of arsenic content in the grain. Rice polishing and alternation in the post-harvest steps (ex; parboiling) could reduce arsenic content in the rice grain (Rahman et al. 2019b; Sandhi et al. 2017). There are several procedures and recommendations for reducing arsenic content in the rice grain at the consumer’s level also which considered proper washing, watering, and pouring excess water after cooking could decrease the level of arsenic exposure through rice consumption in human body (Yang et al. 2017).

## Past and present arsenic management, policies, and practices in Bangladesh

After the first groundwater arsenic contamination in Bangladesh was discovered in 1993, the GOB initiated the first action plan for arsenic management in 1997. They conducted a survey in previously identified arsenic-contaminated areas and found 62% of 32,651 tubewells in 200 villages have arsenic concentrations above 100 µg/L (Smith et al. 2000). During 1999–2005, the GOB launched a program named BAMWSP (Bangladesh Arsenic Mitigation Water Supply Program) financed by World Bank and screenend (blanket survey) As concentrations in tubewells of 270 uppzillas and stored 4.8 million well arsenic concentration data-points in NAMIC (National Arsenic Mitigation Information Centre) (Johnston and Sarker 2007; Balasubramanya and Horbulyk 2018).). Approximately 29.1% of those tested tubewells had arsenic concentrations higher than the Bangladesh standard for arsenic content in drinking water (50 µg/L or 50 ppb) (Kabir and Howard 2007). The national arsenic content tubewell survey was also conducted in 2009 by BBS (Bangladesh Bureau of Statistics) and UNICEF, which found 53 million and 22 million people of Bangladesh were exposed to arsenic contamination according to Bangladesh drinking water standard (50 µg/L) and WHO standard (10 µg/L) respectively (Hossain et al. 2015). During 2004–2010, the DPHE initiated another project named BWSPP (Bangladesh Water Supply Program Project) financed by the World Bank (55.11 million US dollars) for achieving water sanitation-related Millennium Development Goals (MDGs) within 2015 (Tuinhof and Kemper 2010; BWSPP 2013). That project included several components including private sector participation for rural piped water supply, promoted private sector involvement in the municipalities, arsenic mitigation measures in arsenic affected areas, monitoring, and evaluation and capacity building for piped water supply system in the rural areas (BWSPP 2013). Another initiative was also taken by the DPHE, financed by the World Bank (2012–2017) named BRWSSP (Bangladesh Rural Water Supply and Sanitation Project) to increase safe provision of arsenic safe water and hygienic sanitation in the rural areas of Bangladesh and facilitate emergency response (including installation of piped water system in arsenic affected rural areas) (IEG Review Team 2018). The installation of a piped water system for arsenic mitigation options for drinking water demands more focus as the intermediate aquifer has been greatly affected by the pumping process and it has impacts on arsenic contamination in the groundwater of that particular area (Mozumder et al. 2020a). Besides rural locations, arsenic exposure is also increasing in the mega cities of Bangladesh. A recent study has also reported that pumping groundwater in the capital city Dhaka enhances influx of reactive carbon and that increases Fe oxide reduction and arsenic release processes (Mozumder et al. 2020b; Mihajlov et al. 2020). Meanwhile, the feasibility of arsenic mitigation technologies in particular and its profitability also greatly depend on the number of families that could utilize the systems, the water delivery systems, and local employment generation through those technologies (German et al. 2019).

In addition to these large initiatives from the GOB, a number of foreign donors together with different local govt. organizations also implemented several projects for arsenic mitigation in this region. For instance, the Danish International Development Agency (DANIDA) with the DPHE have implemented 6000 deep tubewells in the severely arsenic affected 11 upzillas (2001–2004) (Sharma and Tjell 2003). Similarly, the Swedish International Development Cooperation Agency (Sida) also performed two regional evaluation and monitoring projects named arsenic in tubewell water and health consequences in Matlab Upazila of Chandpur district (AsMat) and sustainable arsenic mitigation (SASMIT) (Hossain et al. 2015). Besides those two agencies, Swiss Agency for Development and Cooperation (SDC), Department of International Development, UK (DFID), Japan Intentional Cooperation Agency (JICA), Swiss Red Cross, United Nations Development Programme, Water Aid, UNICEF, and WHO also conducted a number of arsenic mitigation projects in different regions with local government and NGO organizations (NAMIC 2004). Meanwhile, the number of As mitigation plans and policies in Bangladesh has been declined because of the lack of available foreign aid (Adams 2013). A recent survey conducted by BBS and UNICEF have found that approximately 11.8% population of Bangladesh still frequently use As-contaminated water (> 50 µg/L) as their drinking water sources (BBS and Unicef 2019). Meanwhile, the arsenic testing kits for arsenic measurement in field condition also need to be carefully selected because some of them have been shown to lack of accuracy and precision (Reddy et al. 2020). The habits of people also play an important role for implementation and adaption of new arsenic mitigation technologies as a comparative survey study has found that people are still attached to shallow tubewell water sources even with arsenic or salinity content due to their habit and reliability (Naus et al. 2020).

## Conclusions and outlook

Arsenic-rich drinking water sources were considered to be the most significant environmental health complication in Bangladesh. Regardless of gender, high arsenic exposure in the drinking water increases mortality among young adults (Rahman et al. 2019b). During the last two decades, a number of national and international NGOs alongside government organizations have been working in Bangladesh for investigations, surveys, and arsenic mitigation plan implementation in different parts of this country. Arsenic contamination in the groundwater is not considered a local or regional environmental disaster but rather a global problem. Arsenic concentration in the groundwater is exceeded the WHO maximum permissible limit for drinking water (10 µg/L) in 108 countries globally (Shaji et al. 2021). Apart from developing countries like Bangladesh, developed countries like the USA and Canada also reported As contamination in their groundwater systems (Sorg et al. 2014). This review would provide (1) brief information about case studies for As mitigation plans in a developing country and (2) relative success rates by applying different agricultural managements for As reduction in crops in a similar agro-ecological zone. According to SDG (sustainable development goals) targets announced by UN (United Nations), by 2030, the improvement of water quality and integrated water resources management are considered to be one of the major targets (target 6.3 and 6.5) that need to be fulfilled under this SDGs action plan (UN General Assembly 2015). In conclusion the “take home” message from this review that the development of affordable and sustainable technologies will not be successful for arsenic mitigation strategies in Bangladesh, unless there is an introduction of nationwide arsenic health affect related social awareness schemes on a mass scale.

## Data Availability

Not applicable.
